# Rotavirus vaccination and intussusception – Science, surveillance, and safety: A review of evidence and recommendations for future research priorities in low and middle income countries

**DOI:** 10.1080/21645515.2016.1197452

**Published:** 2016-06-20

**Authors:** Catherine Yen, Kelly Healy, Jacqueline E. Tate, Umesh D. Parashar, Julie Bines, Kathleen Neuzil, Mathuram Santosham, A. Duncan Steele

**Affiliations:** a Division of Viral Diseases, Centers for Disease Control and Prevention, Atlanta, GA, USA; b Johns Hopkins University Bloomberg School of Public Health, Baltimore, MD, USA; c Murdoch Childrens Research Institute, The University of Melbourne, Victoria, Australia; d University of Maryland School of Medicine, Baltimore, MD, USA; e Enteric and Diarrhoeal Diseases, Global Health, Bill and Melinda Gates Foundation, Seattle, WA, USA

**Keywords:** infants, intussusception, rotavirus, rotavirus vaccine, vaccine

## Abstract

As of January 2016, 80 countries have introduced rotavirus vaccines into their national immunization programs. Many have documented significant declines in rotavirus-specific and all-cause diarrheal illnesses following vaccine introduction. Two globally licensed rotavirus vaccines have been associated with a low risk of intussusception in several studies. In July 2014, the Rotavirus Organization of Technical Allies Council convened a meeting of research and advocacy organizations, public health experts, funders, and vaccine manufacturers to discuss post-marketing intussusception surveillance and rotavirus vaccine impact data. Meeting objectives were to evaluate updated data, identify and prioritize research gaps, discuss best practices for intussusception monitoring in lower-income settings and risk communication, and provide insight to country-level stakeholders on best practices for intussusception monitoring and communication. Meeting participants agreed with statements from expert bodies that the benefits of vaccination with currently available rotavirus vaccines outweigh the low risk of vaccination-associated intussusception. However, further research is needed to better understand the relationship of intussusception to wild-type rotavirus and rotavirus vaccines and delineate potential etiologies and mechanisms of intussusception. Additionally, evidence from research and post-licensure evaluations should be presented with evidence of the benefits of vaccination to best inform policymakers deciding on vaccine introduction or vaccination program sustainability.

## Introduction

In 2009, the World Health Organization (WHO) recommended “that rotavirus vaccine for infants should be included in all national immunization programmes, [and] in countries where diarrheal deaths account for ≥ 10% of mortality among children aged < 5 years, the introduction of the vaccine is strongly recommended.”^1,2^ Two live, attenuated, oral rotavirus vaccines − Rotarix (monovalent rotavirus vaccine [RV1], GSK Biologicals, Rixensart, Belgium) and RotaTeq (pentavalent rotavirus vaccine [RV5], Merck & Co. Inc., Kenilworth, NJ) − are WHO-prequalified and currently licensed in > 100 countries. As of 1 May 2016, 81 countries, including 38 low-income countries eligible for support from Gavi, the Vaccine Alliance, have introduced these vaccines into their national immunization programs.^3^ Many of these countries have documented significant declines in rotavirus-specific and diarrheal illnesses following vaccine introduction.^4-14^ In some high- and lower middle-income countries, rotavirus vaccination has been shown to reduce hospitalizations among children and adults who are too old to be vaccinated, an effect known as herd immunity.^14-17^

Adverse events that occur following immunization may or may not be caused by the immunization; if a thorough investigation determines that a causal association exists, then the risk of the adverse event must be considered within the context of the benefits of vaccination. In 1999, the first licensed rotavirus vaccine, RotaShield [RRV-TV] (Wyeth), was withdrawn from the market in the United States (US) due to an elevated risk for intussusception estimated at 1 excess case per 10,000 vaccinated infants. Intussusception is a rare intestinal condition that can cause bowel obstruction.^18-20^ The first consequence of the RotaShield experience was the US Food and Drug Administration's (FDA) requirement to perform large clinical trials prior to the licensure of the new rotavirus vaccines (RV1 and RV5), addressing specifically the intussusception concern with a sample size sufficient to rule out an effect of similar magnitude to that found for RotaShield.^21,22^ Also, during pre-licensure processes, FDA requested large post-licensure safety studies to evaluate the potential risk of intussusception. Other regulatory authorities requested similar commitments. Subsequently, WHO advised post-introduction safety monitoring of the currently licensed rotavirus vaccines.^2^ Although RV1 and RV5 were not associated with intussusception during large pre-licensure clinical trials for each of > 60–70,000 infants, early post-marketing surveillance indicated a small increased risk of intussusception, primarily shortly after the first dose of vaccination, with both rotavirus vaccines in some populations, but not others.^23-30^

In March 2011, PATH convened a meeting of technical experts and public health officials to review emerging data on intussusception related to these 2 globally available rotavirus vaccines, establish what knowledge gaps existed, and identify future research needs.^31^ Participants from this meeting recognized that, although a small risk of intussusception had been detected in some settings following vaccination − mostly with the first dose of both licensed rotavirus vaccines (∼1–6 excess cases per 100,000 vaccinated infants) − this was lower than the risk with RRV-TV, and the documented benefits of rotavirus vaccination were substantial and well-documented and judged by the group to outweigh the risks.^31,32^ Future priority activities for intussusception and rotavirus vaccines identified at this meeting were related to preparing for future vaccine introduction in lower-income settings of Africa and Asia, addressing gaps in knowledge, addressing and monitoring issues related to strict age recommendations, and developing key messages for communication about intussusception (Table 1).

**Table 1.** Future Priorities for Rotavirus Vaccines and Intussusception Monitoring - Recommendations from the Rotavirus Vaccination & Intussusception Workshop, July 2014.

Key messages for risk communications • Ensure that stakeholders are aware that robust surveillance has demonstrated consistent, strong benefits of rotavirus vaccination, and that risks associated with rotavirus vaccine, including intussusception, remain very low in all post-licensure safety studies to date. Present information about baseline risk of intussusception. Share information with all levels of decision-makers in countries considering vaccine introduction.
Vaccine introduction and sustainability in low- and middle-income countries • Integrate safety monitoring for rotavirus vaccines into routine vaccine safety monitoring: ○ Ensure that countries have clear risk management and communications plans for adverse events following immunization ˆ *Conduct targeted safety monitoring while strengthening capacity for routine vaccine safety monitoring* • Ensure that, wherever possible, vaccine impact evaluations occur alongside safety monitoring to allow comprehensive assessment of potential risks within the context of benefits: ˆ *Leverage existing surveillance networks to increase efficiency* • Understand the regional epidemiology of intussusception to allow accurate interpretation of findings from safety monitoring within the context of comparable data concerning benefit: ˆ *Establish background rates and outcomes of intussusception in select areas for each region* ˆ *Focus on low- and middle-income countries for which there are limited data* • Examine the risk of intussusception associated with vaccination in early introducer, regionally representative countries using feasible methods: ˆ *Ensure that findings are shared broadly to inform the experiences of other countries* • Document use, benefit, and safety of rotavirus vaccines in countries that have implemented the expanded age recommendations for rotavirus vaccine administration.
Gaps in knowledge • Support research to increase understanding of the pathogenesis and etiology of intussusception. This would include the following areas: ˆ Basic science studies to understand better the pathogenesis of and triggers for idiopathic intussusception in young children ˆ Epidemiological studies to understand the occurrence of transient (i.e., self-resolving) and persistent idiopathic intussusception in different regions and settings: – *Standardize case definitions and methodologies to allow pooling of data* – *Share findings with all stakeholders in a timely manner* • Explore why some studies have detected an increased risk of intussusception following the first dose of vaccine and other studies have not: ˆ Role of co-administration of inactivated and oral polio vaccines ˆ Differences in immune response and shedding across populations • Support studies to determine risk-benefit assessments for countries with higher baseline rates of intussusception.
Evaluation of new rotavirus vaccines in development • Conduct post-marketing surveillance for intussusception in appropriate representative sites also conducting vaccine impact evaluations. • Continue to monitor the potential association of specific vaccine strains with adverse events following immunization post-licensure.

In April 2012, the WHO Strategic Advisory Group of Experts on Immunization (SAGE) met to review new evidence on rotavirus vaccines, including the following: age-specific burden of rotavirus diarrhea and deaths, timeliness of vaccine administration, vaccine safety, and effectiveness of various immunization schedules.^2,32,33^ As a result, the age restrictions on the first and last doses of rotavirus vaccines were removed from the WHO-recommended immunization schedule in order to reach children who previously would not have received rotavirus vaccines due to delayed presentation for health care. Additionally, more information on intussusception with currently licensed rotavirus vaccines, as well as new evidence on health benefits associated with rotavirus vaccine have become available;^5,8-10,12,15,16,23-25,29,30,34-36^ new rotavirus vaccines have been licensed in some countries (Vietnam, China, India), and others are in late stages of clinical testing.

Given this, the Rotavirus Organization of Technical Allies (ROTA) Council and its Core Partners, the US Centers for Disease Control and Prevention (CDC), Johns Hopkins Bloomberg School of Public Health, PATH, and the Sabin Vaccine Institute, convened the Rotavirus Vaccination & Intussusception Workshop in July 2014. This workshop was a follow-up meeting of the 2011 intussusception meeting of technical and public health experts.^a^ aThis workshop was organized by the ROTA Council and held on 23-24 July 2014 in Washington, DC. It included invited representatives from the following institutions, organizations, and companies: Baylor University, Houston, TX, USA; Christian Medical College, Vellore, India; INCLEN Trust, New Delhi, India; Johns Hopkins Bloomberg School of Public Health, Baltimore, MD, USA; Stanford University, Stanford, CA, USA; University of Melbourne, Melbourne, Australia; Yale University, New Haven, CT, USA; the Centers for Disease Control and Prevention, Atlanta, GA, USA; the Bill and Melinda Gates Foundation, Seattle, WA, USA; Gavi, the Vaccine Alliance, Geneva, Switzerland; the National Institutes of Health, Bethesda, MD, USA; PATH, Seattle, WA, and Washington, DC, USA; the Sabin Vaccine Institute, Washington, DC, USA; the World Health Organization, Geneva, Switzerland; GlaxoSmithKline Biologicals, Rixensart, Belgium; Merck Vaccines, West Point, PA, USA; Serum Institute of India, Pune, India; and Shantha Biotechnics, Hyderabad, India. The objectives of the meeting were to 1) review and evaluate the current evidence on intussusception and rotavirus vaccines, including emerging information, recent recommendations by global bodies, and early data on vaccine products in various stages of development/licensure; 2) identify and prioritize remaining gaps in the research; 3) achieve consensus on best practices for intussusception monitoring in lower income settings – pre- and post-introduction; 4) demonstrate best practices for communicating about risk; and 5) provide insight to country-level stakeholders on best practices for monitoring and communicating about intussusception.

In this paper, we provide a review of evidence on rotavirus vaccine-associated intussusception and statements from expert bodies regarding the benefits and risks of rotavirus vaccination and present main discussions and recommendations that arose from the July 2014 meeting.

## Intussusception

Intussusception is a rare condition in which the intestine folds in on itself.^37,38^ Although many cases of intussusception self-resolve, others result in bowel obstruction that can be fatal if not treated promptly. Treatment for intussusception includes reduction by air, hydrostatic enema, or surgery. Intussusception is most likely to occur naturally in infants, in the absence of vaccination, between 4 and 10 months of age. The mean incidence is ∼74 cases per 100,000 infants < 1 y of age (range: 9-328), but is variable across geographic regions, most notably with higher rates in parts of Asia.^39,40^ Reasons for regional variation are not well defined, but could relate to multiple factors including genetic predisposition, circulating pathogens, differences in feeding practices, and differences in diagnostic practices and access to health care.^40^ The cause of intussusception in the majority of infants is not known. Some infectious agents, particularly respiratory adenoviruses and pathogens causing bacterial enteritis (e.g., *Campylobacter* spp., *Escherichia coli, Salmonella* spp., and *Yersinia enterocolitica*), have been temporally associated with intussusception in some studies.^39,41-44^ Additionally, the mechanism(s) of intussusception in children are not known, but are postulated to include lymphoid hyperplasia causing lead points, inflammation in the intestine, or alterations in gastrointestinal motility.^38,45,46^

## Intussusception and rotavirus vaccines – a review of the evidence

### RRV-TV

RRV-TV (Wyeth), a 3-dose, oral tetravalent rhesus-human reassortant vaccine, administered within the first year of life, was the first rotavirus vaccine licensed and was recommended for use in the US. During pre-licensure clinical trials, vaccine efficacy was estimated to be 50–70% against rotavirus disease of any severity and 70–90% against severe disease;^47^ fever, irritability, and abdominal cramping were common among vaccine recipients during the first 3–5 d following vaccination. These trials of RRV-TV were not designed to assess a risk of intussusception. However, there were a few intussusception reports among vaccine recipients, and though there was not a statistically significant risk associated with the vaccine, monitoring of this adverse event post-licensure was recommended.^19,48^ Intussusception was included in the vaccine package insert as a possible adverse event, as well as in the Advisory Committee on Immunization Practices (ACIP) and American Academy of Pediatrics (AAP) recommendations.^47,49^ A search term for intussusception was also created for the passive Vaccine Adverse Event Surveillance System (VAERS). Less than one year after introduction, rotavirus vaccination was temporarily suspended due to 15 case reports in VAERS of intussusception among infants who had received RRV-TV.^18^ A subsequent national case-control study found an increased risk of intussusception 3 to 14 d following the first dose of RRV-TV (adjusted odds ratio, 21.7, 95% confidence interval [CI]: 9.6–48.9) and a smaller risk (adjusted odds ratio, 3.3, 1.1–9.8 95%CI) following the second dose.^19^ The attributable risk was estimated as 1 excess cases of intussusception per every 10,000 vaccinated infants.^20^ Following the availability of these data, the recommendation for RRV-TV use in the US was withdrawn by ACIP. Although many public health experts were supportive of further evaluating RRV-TV use in developing countries, such evaluations did not occur because some developing countries were reluctant to test a product that had been withdrawn from the US market. Lessons learned from this experience included the following: 1) It is difficult to conduct a clinical trial large enough to detect such a rare adverse event; 2) well-designed post-licensure analytical observational studies are critical; and 3) recommendations should be based upon risk-benefit analyses for each region.

### RV1 and RV5

Large pre-licensure clinical trials of 60,000-70,000 infants each for RV1 and RV5 in high and middle income countries demonstrated high efficacy against severe rotavirus disease and no association of intussusception with either vaccine.^26,50^ As these large trials could not exclude a risk lower than that previously observed for RRV-TV of intussusception within a short period after vaccination, post-licensure surveillance studies were initiated in several early vaccine introduction countries: Brazil and Mexico (RV1 only), Australia (RV1 and RV5), and the United States (RV1 and RV5) (Table 2).

**Table 2.** Risk-benefit of rotavirus vaccination on rotavirus hospitalizations and deaths and associated intussusception risk for one vaccinated birth cohort to age 5 years, multiple countries. *While some of these data were not discussed in the meeting, they may help to provide context around the broader issue of intussusception*.

Country [ref]	Vaccine evaluated	Vaccine dose(s)	Overall attributable risk (excess IS cases per 100,000 vaccinated infants)	Rotavirus outcomes averted	IS outcomes caused
Mexico ^23^	RV1	Dose 1 only	2.0–3.7	Hospitalizations: 11,551	Hospitalizations: 41
				Deaths: 663	Deaths: 2
Brazil ^23^	RV1	Dose 2 only	1.5	Hospitalizations: 69,572	Hospitalizations: 55
				Deaths: 640	Deaths: 3
Australia ^50^	RV1	Doses 1 and 2	4.3	Hospitalizations: 6528	Hospitalizations: 14
				Deaths: Not reported	Deaths: Not reported
	RV5	Doses 1 and 2	7.0		
US ^27,28^	RV1	Doses 1 and 2	5.3	Hospitalizations: 53,444	Hospitalizations: 35–166
		Dose 2 only	7.3	Deaths: 14	Deaths: 0.1–0.5
	RV5	Dose 1 only	0.7–1.5	NA	NA

IS = intussusception

**A. *Brazil and Mexico***. RV1 was introduced into the national immunization programs of Brazil in March 2006 and Mexico in May 2007. In one study conducted at 53 hospitals in Brazil and 16 hospitals in Mexico, self-controlled case-series (SCCS) and case-control methods were used to evaluate the risk of intussusception following rotavirus vaccination.^25^ In Mexico, 285 children were enrolled, and 330 were enrolled in Brazil. Among Mexican children, a 5-fold increase in risk of intussusception was observed in the 1–7 d after dose 1 by both the SCCS and case-control analyses. In Brazil, no risk was observed after the first dose of the vaccine, but a smaller, 2-fold increase in risk was observed in the 1–7 d following dose 2. Co-administration with oral polio vaccine, as opposed to co-administration with inactivated polio vaccine which occurred in Mexico, was speculated to explain the absence of risk associated with the first dose of rotavirus vaccine in Brazil since the first dose of oral polio vaccine has been shown to decrease the immunogenicity of the first dose of rotavirus vaccine. However, other factors, such as feeding practices, natural risk of intussusception, and maternal antibody levels, could have contributed as well.^25^ The overall attributable risk in both settings was 1.5–2 excess cases of intussusception per 100,000 vaccinated infants.

A separate study using SCCS methods conducted at 67 hospitals in Mexico found that, of 753 episodes of intussusception reported in 750 infants, 701 were in vaccinated infants, with a statistically significant temporal association between intussusception and RV1 during the 31 d after dose 1 (relative incidence [RI] = 1.75, 95% CI: 1.24–2.48);^51^ this effect was concentrated in the first week (RI = 6.49, 95% CI: 4.17–10.09). The estimated attributable risk was 3.7 excess cases of intussusception per 100,000 vaccinated infants.

**B. *Australia***. Rotavirus vaccination was introduced into the National Immunization Program in July 2007, with each of 8 jurisdictions (6 states and 2 major mainland territories) independently choosing to administer RV1 or RV5 and rapid increases in vaccination coverage was observed following introduction (85%).^52^ An initial post-marketing surveillance study with a reporting period of 1 July 2007 to 31 December 2008 suggested an increased risk of intussusception in the dose 1 post-vaccination risk window.^23^ In response to this signal, the Australian Therapeutic Goods Administration commissioned a national study to evaluate the risk of intussusception following rotavirus vaccination and to compare the vaccine-attributable risk of intussusception with the estimated reduction in gastroenteritis hospitalizations following vaccine introduction. Confirmed cases of intussusception in infants 1 to <12 months were identified from national hospitalization databases, supplemented by active hospital-based surveillance from July 2007 to June 2010. SCCS and case-control methods were used to assess risk of intussusception associated with both vaccines in pre-specified periods post-vaccination. Based on 306 confirmed cases of intussusception, the relative incidence of intussusception in the 1–7 day period following dose 1 was 6.8 (95% CI: 2.4 to 19.0) for RV1, and 9.9 (95% CI 3.7 to 26.4) for RV5.^52^ There was a smaller increased risk 1–7 d following dose 2 of each vaccine. The relative incidence of intussusception for RV1 was 2.84 (95%CI: 1.10 to 7.34) and 2.81 (95%CI: 1.16 to 6.80) for RV5.^52^ The case-control analysis gave similar results. The overall attributable risk for both vaccines was 5.6 excess cases of intussusception per 100,000 infants vaccinated.^52^

**C. *United States***. Universal rotavirus vaccination was again recommended in the United States in 2006, first with RV5, licensed in 2006, then with either RV5 or RV1, licensed in 2008.^53^ Initial analyses of VAERS data, which evaluated passive reporting for February 2006 through September 2007 identified 160 reports of intussusception following RV5 vaccination.^54^ Although comparison of reporting rates with baseline intussusception rates showed no increased risk during the 21-day period following vaccination, the clustering of cases during the first week following vaccination was noted and further monitoring was recommended. Additional early post-licensure studies of RV5 evaluated data from the Vaccine Safety Datalink (VSD). The VSD project is a collaboration between CDC and several integrated health care organizations for which weekly updates of electronic data regarding vaccinations and health care utilization from hospitals, emergency departments, and outpatient clinics are used to conduct real-time surveillance of adverse events following vaccination.^29^ An analysis of data from children aged 4 to 48 weeks enrolled in the VSD who received RV5 between May 2006 and May 2008 did not identify an elevation in risk for intussusception through 30 d after RV5 vaccination.^55^ A subsequent VSD cohort study of children aged 4 to 34 weeks who received RV5 between May 2006 and February 2010 also did not identify an elevation in intussusception risk during 1- to 7-day and 1- to 30-day risk windows following RV5 vaccination.^27^ One other study evaluating data from a large research database of health insurance reimbursement claims also did not identify an elevated risk for intussusception.^56,57^

More recent studies evaluating VAERS and VSD data have identified a low elevated risk of intussusception following vaccination with either RV1 or RV5. An updated analysis using VAERS data for February 2006 through April 2012 identified 584 and 52 reports of intussusception following RV5 and RV1 vaccination, respectively, with clustering of reported intussusception events 3 to 6 d after the dose 1 of RV5 and 4 to 7 d after dose 1 of RV1.^24^ Using self-controlled risk interval analysis, the estimated excess risk of intussusception following vaccination with dose 1 of RV5 was 0.74 cases of intussusception (95% CI: 0.24–1.71) per 100,000 vaccinated infants. A study by the VSD project evaluating the relative risk (RR) of intussusception among children aged 4 to 34 weeks who had received RV1 between April 2008 and March 2013 compared the observed rate of intussusception with the expected background rate generated from historical, pre-rotavirus vaccine introduction rates of intussusception.^29^ This study identified a statistically significant elevated risk of intussusception within 7 d following vaccination with either dose (RR: 8.38) and with dose 2 (RR: 8.17) of RV1; the statistical significance of intussusception risk following vaccination with dose 1 (RR: 8.82) could not be calculated due to the low number of cases.^29^ The increased risk for intussusception following vaccination with RV1 was similar when historical and concurrent chart-confirmed studies were conducted, and the estimated attributable risk was 5.34 cases per 100,000 vaccinated infants. A VSD analysis comparing observed rates with expected rates of intussusception for children aged 4 to 34 weeks who had received RV5 between May 2006 and March 2013 did not identify an elevated risk of intussusception within the 7 d following vaccination with all doses (RR: 1.13, 95% CI: 0.49–2.22), or following vaccination with dose 1 (RR: 2.63, 95% CI: 0.72–6.74), dose 2 (RR: 0 [0 observed cases], 95% CI: 0–1.26), or dose 3 (1.25, 95% CI: 0.34–3.20).^29^

In addition to the VAERS and VSD findings, an analysis of health insurance claims data on 1.3 million doses of RV5 and 100,000 doses of RV1 from the Post-licensure Rapid Immunization Safety Monitoring System (PRISM), part of a Food and Drug Administration-sponsored pilot program to conduct surveillance for medical product safety, identified 124 confirmed cases of intussusception.^30^ A self-controlled risk interval analysis found an increased relative risk of intussusception of 9.1 (95% CI: 2.2–38.6) during days 1 to 7 following dose 1 of RV5, translating to an attributable risk of 1.1 cases per 100,000 vaccinated infants, and a relative risk of 4.2 (95% CI: 1.1.-16) during days 1 to 21 following dose 1 of RV5, translating to 1.5 cases per 100,000 vaccinated infants. A secondary cohort analysis also found an elevated risk of intussusception during days 1–21 following dose 1 of RV5 (RR: 2.6, 95% CI: 1.2–58), translating to an attributable risk of 1.2 cases per 100,000 vaccinated infants. While the self-controlled risk interval analysis did not identify any elevated risk for intussusception following RV1 vaccination, the secondary cohort analysis identified a relative risk of intussusception of 5.1 (95% CI: 1.6–16.4) following dose 2 of RV1, translating to an attributable risk of 7.3 cases per 100,000 vaccinated infants.

## Global benefits of rotavirus vaccination and statements of expert bodies on risk-benefit of vaccination

As some countries have identified a low risk of intussusception with both RV1 and RV5 in post-licensure evaluations, extensive data on the benefits of vaccination have also been reviewed. A systematic review of data from 8 countries reported a 49%-89% decline in laboratory-confirmed rotavirus hospitalisations and 17%-55% decline in all-cause gastroenteritis hospitalisations among children <5 y within 2 y of vaccine introduction.^58^ As an unanticipated benefit, in some countries, rotavirus vaccination of young infants has also resulted in the declines in rotavirus disease among children who missed vaccination and among older children and even adults who were not vaccine-eligible, a phenomenon known as herd immunity.^14-17^ Herd immunity is likely related to reduction in community transmission of rotavirus because of vaccination. Notably, studies from Mexico and Brazil showed a reduction in childhood deaths from diarrhea following vaccine implementation, a key outcome that was not evaluated in clinical trials.^7,59^

Based on these data, key stakeholders and technical experts at national and global levels of policymaking have reviewed the risks of intussusception in conjunction with the demonstrated benefits of rotavirus vaccination (Table 2). Policymakers in Australia, Brazil, Mexico, and the United States have concluded that the benefits of rotavirus vaccination far outweigh the risks of intussusception and continue to recommend rotavirus vaccination in their countries.^32,60-63^ Additionally, the Global Advisory Committee on Vaccine Safety (GACVS) of WHO has acknowledged the risk of intussusception following the current rotavirus vaccines, but has also concluded that the risk of intussusception remains small in comparison to the benefits of preventing severe diarrhea.^64^ WHO continues to recommend that rotavirus vaccination be included in all national immunization programs.^2^

## Age restrictions on rotavirus vaccination

As the risk-benefit of intussusception and rotavirus vaccines continues to be monitored, a related discussion regarding age restrictions for rotavirus vaccination has occurred. Based on data from the RRV-TV investigations that suggested a potentially higher risk of intussusception among children vaccinated at an older age (i.e., >3 months), WHO, in its initial rotavirus vaccine position paper endorsed by SAGE, recommended the inclusion of age restrictions for vaccination with RV1 and RV5 –the first dose was to be given no later than 15 weeks of age, and the last dose no later than 32 weeks.^1^ However, in June 2009, GACVS reviewed the available data and determined the data were insufficient to conclude whether the risk of intussusception differed by age in children who were vaccinated with RRV-TV.^65^ Because rotavirus vaccine is administered together with the diphtheria-tetanus-pertussis (DTP) vaccine and given the imposed age restrictions, this would likely result in the exclusion of a substantial number of children who present late for vaccinations, particularly in developing countries. In developed countries, vaccination is timelier, so there would not be substantial added value in relaxing the age restriction. An analysis was undertaken to examine the potential risk-benefit of rotavirus vaccination without age restrictions.^33^ This analysis showed that universal rotavirus vaccination in low- and low-middle-income countries could prevent an additional 47,200 (range: 18,000–63,700) rotavirus deaths while potentially causing an additional 294 (range: 161–471) intussusception deaths among a cohort of children <5 y of age. In April 2012, SAGE reviewed these data and determined that removal of the age restrictions was programmatically easier, and would result in additional lives saved that would far outnumber the excess vaccine-associated intussusception cases and deaths.^66^ This led to the 2013 WHO recommendation for removal of upper age restrictions for rotavirus vaccination to allow for greater vaccination coverage and potentially greater impact on preventing rotavirus-related deaths in developing countries.^2^ WHO continues to recommend administering the first dose of rotavirus vaccine as soon as possible after 6 weeks of age, along with DTP1 vaccination, and recommends that countries develop a strategy to inform health staff that the benefits of vaccination outweigh the small potential risk of intussusception.

## Post-vaccine introduction surveillance in low and middle income countries

Several activities to assess the safety of rotavirus vaccines are planned or are in progress; such studies are needed to conduct risk-benefit analyses. These assessments are taking place in several representative countries that will also evaluate the impact and effectiveness of rotavirus vaccine to ensure that risk and benefit data are obtained from the same settings. These studies have been supported by organizations such as Gavi, the Vaccine Alliance, and the Bill & Melinda Gates Foundation. Key evaluations to monitor rotavirus vaccines are being conducted in Africa. Between 2009 and 2014, rotavirus vaccines were introduced into the national immunization programs of 25 African countries – over half of which introduced a rotavirus vaccine during 2013 and the first half of 2014. A few countries conducted surveillance for intussusception prior to vaccine introduction, and these surveillance studies have provided some key insights into the epidemiology of intussusception in Africa.^67-69^ In 2013, an evaluation began in South Africa to assess rotavirus vaccine safety after routine introduction in 2009. This study has recruited pediatric hospitals across the country as part of its active intussusception surveillance network. Multi-country post-marketing evaluations designed to evaluate the association of rotavirus vaccination and intussusception are also ongoing at major pediatric hospitals throughout Africa.

## Review of past priorities, remaining research questions, and future priorities

In reviewing old and new data regarding intussusception and rotavirus vaccination, participants of the July 2014 meeting agreed with the statements of expert bodies that the benefits of rotavirus vaccination continue to significantly outweigh the risks of vaccine-associated intussusception.^31,64,65^ The participants also strongly believe that any discussion of intussusception and rotavirus vaccines must occur alongside discussion of the benefits of vaccination. In other words, discussion of the risks *and* benefits of rotavirus vaccination, not just risks, should always occur. The following past priorities, remaining research questions, and future priorities are those discussed and recommended by the meeting participants.

- ***Past priorities***: In reviewing the priorities set during the March 2011 meeting, it was thought that a broader focus on ***vaccine introduction and sustainability in low- and middle-income countries*** should be taken to:
  - Ensure stakeholders are aware that robust surveillance has demonstrated consistently the benefits of rotavirus vaccination, and that risks associated with rotavirus vaccine, including intussusception, remain very low in all post-licensure safety studies to date.
  - Integrate safety monitoring for rotavirus vaccines into routine vaccine safety monitoring. However, at a time when countries are working to establish or improve basic routine adverse events reporting systems, the most efficient way to achieve this is through well designed, post-licensure evaluations using case-control or case-only methods such as self-controlled case series methods.
    - Ensure that countries have clear risk management and communications plans for adverse events following immunization.
    - Conduct targeted safety monitoring while strengthening capacity for routine vaccine safety monitoring.
  - Ensure that, wherever possible, vaccine impact evaluations occur alongside safety monitoring to allow comprehensive assessment of potential risks within the context of benefits.
    - Leverage existing surveillance networks to increase efficiency.
  - Understand the regional epidemiology of intussusception to allow accurate interpretation of findings from safety monitoring within the context of comparable data concerning benefit.
    - Establish background rates and outcomes of intussusception in select areas for each region.
    - Focus on low- and middle-income countries for which there are limited data, where the risk profile for intussusception may differ due to lower efficacy estimates compared to higher income settings, as demonstrated in clinical trials.^70,71^
    - Examine the risk of intussusception associated with vaccination in early-introducer, regionally representative countries using feasible methods.
    - Ensure that findings are shared broadly to inform the experiences of other countries
  Past priorities identified for addressing and monitoring issues related to strict ***age recommendations*** have been addressed. Therefore, it is now time to document use, benefit, and safety of rotavirus vaccines in countries that have implemented the expanded age recommendations for rotavirus vaccine administration.
- ***Remaining research questions***: Mouse models provided the first, direct experimental proof that rotavirus can contribute to the development of intussusception, independent of strain type and provided evidence that viral replication is required to induce intussusception, as live, but not inactivated, rhesus rotavirus vaccine (RRV) significantly enhanced intussusception rates in mice;^72^ although, systemic replication capacity varies between strains and is not necessarily host range restricted.^73^ Based on these data, researchers predicted that any live rotavirus vaccine could cause intussusception, but that inactivated or subunit vaccines would not. The identification that the second-generation rotavirus vaccines, RV5 and RV1, have also been associated with a low-level excess risk of intussusception in some vaccinated children validated these predictions.
  Regarding potential mechanisms of intussusception, mouse models have demonstrated that, despite the induction of massive intestinal lymphoid hyperplasia following wild-type rotavirus infection, lymphoid hyperplasia is not required as a lead point for rotavirus-induced intussusception, and rotavirus infection does not directly alter intestinal motility to induce intussusception.^74^ More recently, a mouse model of inflammation-induced intussusception provided the first experimental identification of a mechanism of intussusception in which lipopolysaccharide (LPS)-induced intussusception was mediated by innate immunity factors, including toll-like receptor 4 (TLR4) and phagocytes, and by hypo-contractility of the intestine.^75^ These studies have provided useful data for further exploring and defining mechanisms of intussusception.
  Further research is also needed to increase understanding of the ***pathogenesis and etiology of intussusception***. Such research should include:
  - Basic science studies to better understand the pathogenesis of, and triggers for, idiopathic intussusception in young children
  - Epidemiological studies to understand the occurrence of transient (i.e., self-resolving) and persistent idiopathic intussusception in different regions and settings
    - Standardize case definitions and methodologies to allow pooling of data
    - Share findings with all stakeholders in a timely manner
  - Studies that determine risk-benefit assessments for rotavirus vaccination among countries with higher baseline rates of intussusception
  Further research is needed to understand how rotavirus and intussusception may be related. Such research should:
  - Understand better the mechanisms of intussusception in humans
  - Assess the role of infectious agents, genetics, and microbiome in intussusception
- ***Future priorities***: Clinical efficacy trials for newer oral rotavirus vaccines continue to evaluate the potential for intussusception, although sample sizes are ∼7,000 infants, and intussusception rates similar to RV1 or RV5 will only be determined in postmarketing surveillance. The most recent published data are from the phase III clinical trial for the recently licensed vaccine ROTAVAC (Bharat Biotech International, India). ROTAVAC is a 3-dose, live, attenuated oral vaccine containing a naturally occurring human-bovine reassortant G9P[11] strain, also known as the 116E strain. A clinical trial including ∼7,000 Indian infants demonstrated 56.4% efficacy against severe rotavirus gastroenteritis during the first year of life.^76^ ROTAVAC was not associated with any serious adverse events, including intussusception, in the phase III trial. Six (< 1%) cases of intussusception were reported in the vaccine group and 2 (< 1%) were reported in the placebo group (with a 2:1 allocation of vaccine and placebo recipients). All events took place after administration of dose 3. The minimum interval between dosing and intussusception was 112 d in the vaccine group and 36 d in the placebo group.^76^ ROTAVAC has been licensed for use in India in 2014 and has been recommended for inclusion in the Universal Immunization Program of India. Post-marketing assessment of intussusception with ROTAVAC will be conducted.

Additional unpublished information was discussed for several other vaccines in development, including a bovine pentavalent rotavirus vaccine (BRV-PV, Serum Institute of India); a bovine-human reassortant tetravalent rotavirus vaccine (Shantha Biotechnics, India) that has recently entered a phase III clinical trial; and a natural human neonatal G3P[6] strain vaccine (RV3, MCRI, Melbourne, Australia) which is currently in phase II trials in Indonesia. Limited data on intussusception exist for these vaccines from the relatively small trials to date.

Given an urgent need for new rotavirus vaccines, it would acceptable for vaccine manufacturers of newer rotavirus vaccines with a similar profile to that of current vaccines to conduct pre-licensure safety and efficacy studies of approximately 5,000–7,000 infants, however post-licensure studies would be required to evaluate a rare event such as intussusception.

Since it is impractical for emerging manufacturers to power pre-licensure studies to detect rare adverse events, ***post-marketing surveillance should be established*** to:

• Document impact and rare adverse events, including intussusception, associated with new rotavirus vaccines specifically developed for use in lower income settings. These assessments should be conducted in select sentinel sites also conducting vaccine impact evaluations.

• Continue to monitor through analytical observational studies the potential association of specific vaccine strains with adverse events following immunization post-licensure. Where regional data are available, it is not necessary to have every country establish post-marketing surveillance for rare events as this will not be feasible for every country to undertake.

## Summary

Expert bodies have concluded that the benefits of vaccination with the current globally available rotavirus vaccines substantially outweigh the low risk of intussusception associated with vaccination.^31,64,65^ Although safety monitoring for rotavirus vaccines should ideally be routinely implemented, current systems in lower income settings are non-existent or insufficient, and AEFI systems should be strengthened. Furthermore, well-designed post-licensure evaluations should be conducted in regionally representative countries that are also conducting impact evaluations of rotavirus vaccination. To better understand the relationship of intussusception to wild-type rotavirus (and rotavirus vaccines), research is needed to further delineate the potential etiologies and mechanisms of intussusception. Evidence generated from research and post-licensure evaluations should be presented within the context of evidence of the benefits of vaccination to best inform policymakers deciding on vaccine introduction or sustainability of their vaccination programs. Appropriate strategies should be developed to communicate that the benefits far outweigh the risks of intussusception based on data available to date.

## Disclaimer

The finding and conclusions of this report are those of the authors and do not necessarily represent the official position of the US Centers for Disease Control and Prevention.
